# Internet-based Patient Self-care: The Next Generation of Health Care Delivery

**DOI:** 10.2196/jmir.5.2.e8

**Published:** 2003-05-15

**Authors:** June Forkner-Dunn

**Affiliations:** ^1^Kaiser Foundation Health Plan, IncInternet Services GroupOakland CaliforniaUSA

**Keywords:** Consumer participation, Internet, medical informatics, patient education, physician-patient relations, self care, technology, disease management, computers

## Abstract

The United States health care system is an outdated model in need of fundamental change. As part of this change, the system must explore and take advantage of the potential benefits of the "e-revolution," a phenomenon that includes everyday use of the Internet by the general public. During 2002, an estimated 100 million Americans will have obtained information — including health information — from the Web as a basis for making decisions. The Internet is thus an influential force; and, as such, this medium could have a revolutionary role in retooling the trillion-dollar United States health care industry to improve patient self-management, patient satisfaction, and health outcomes. As a group, physicians use the Internet more than do many other sectors of the general adult population. However, physicians have not received sufficient information to convince them that they can provide higher-quality care by using the Internet; indeed, few studies have assessed the Internet's value for improving patients' medical self-management and health behavior, as well as their clinical outcomes and relationships with health care practitioners. New e-technology formats introduced to the growing consumer movement will drive the next generation of self-care by allowing patients to manage their own health conveniently and proficiently.

## Introduction

The landmark Institute of Medicine report, "Crossing the quality chasm: a new health care system for the 21st century" [1], depicts an outdated model of health care that hosts worsening chronic medical conditions, skyrocketing health care expenditures, and failure to effectively transform technical innovation into improved health outcomes. The Internet may have a revolutionary role in retooling the trillion-dollar health care industry in the United States.

Indeed, by introducing new e-technology formats to the growing consumer movement, the online revolution may become the engine driving the next generation of self-care, thereby allowing patients to manage their own health conveniently and proficiently. Although the Internet's power to positively affect care management seems an intuitive concept, the Internet's value for improving health outcomes must be examined and documented to provide a basis for further advancement.

## The Online Revolution

Public use of the Internet as a health care tool has grown dramatically in the past few years, and this trend is expected to continue. During 2002, more than 100 million Americans will have searched online for information, including health information — an increase of 13 million from the previous year [2]. Obtaining information from the Web is often the basis for making health decisions and is thus an influential force. Of persons surveyed in 2000 by the Pew Internet & American Life Project, 41% said that the Internet affected their decisions about going to a doctor, treating an illness, or questioning their doctor [3].

This online phenomenon is occurring while a huge population segment, the postwar "baby boomers," is moving like a tsunami through the American health care system. Thanks to modern medicine, these adults will live longer than earlier generations ever could — and will flood the health care system with chronic ailments. Moreover, in addition to making health care decisions for themselves, this population is making such decisions for their elderly parents, many of whom have multiple chronic diseases. Baby boomers are demanding the same easy access to advanced health care technology as is currently available to them when they do their banking or plan a vacation. We have arrived at the era of the "impatient patient." Patients demand immediate, convenient access to a high level of personalized health care: they want it their way, and they want it now.

## Effect of e-Technology on the Patient-Physician Relationship

Can the Internet empower patients? Can it enrich the patient-physician relationship? Breast cancer patients in an online education and support group had increased confidence in their physicians, as well as increased competence to deal with relevant, disease-related information. These patients were also more comfortable seeking information during a physician office visit and were more comfortable participating in their own care [4]. This study alone is minimal evidence to support changes in the patient-physician relationship and more research is needed.

A Harris Online poll found that patients who use the Internet to look for health information are more likely to ask more specific and informed questions of their doctors and to comply with prescribed treatment plans [5]. This was a survey and not a formal study. Further research is necessary to understand what effect the Internet age has on the patient-physician relationship. For example, are patients more compliant with prescribed therapy because they discussed it more with their physician or because they read it on the Web?

The "school of lay medicine" found on the Internet offers an important opportunity for patients to become actively engaged in their own care. During the pre-Internet era, medical information was published in medical textbooks and journals only, whereas patients can now gain access to citations of more than 12 million medical articles online [6]. Indeed, many patients are now helping to inform their physicians on the latest research and treatments.

Physicians Gerber and Eiser [7] postulate that the Internet age offers opportunities to improve the patient-physician relationship by sharing the burden of responsibility for knowledge. They also underline the necessity for research to identify the effects on the patient-physician relationship, as well as the effects on patient and physician satisfaction and on health outcomes.

The locus of power in health care also is shifting: instead of the doctor acting as sole manager of patient care (ie, "the captain of the ship"), a consumerist model has emerged in which patients and their doctors are partners in managing the patient's care [8]. On the other hand, there are many patients who do not wish to be the captain of the ship. Research needs to address how the Internet would affect these individuals.

## Patient Self-management Using e-Monitoring

Several monitoring devices using the Internet have been developed to help patients manage their medical conditions at home. For example, diabetic patients can test their blood glucose level by using an e-device, which with the click of a computer mouse downloads the result to a health care practitioner. Patients with heart failure can step on an e-scale, which sends instantaneous alerts to health care professionals when the patient's weight exceeds the desired range. An e-shirt can be worn which transmits heart rate and respiratory rate over the Internet. A pill-sized camera can be swallowed which transmits pictures of the digestive tract over the Internet. Research is needed regarding health outcomes, cost effectiveness, as well as the long-term acceptance of these devices by patients.

The federal government has invested $28 million to evaluate home glucose monitoring via the Internet to homes of underserved rural and inner-city residents in New York State. The largest eHealth grant ever funded by the government, this study will serve as an important test case for the possibility of e-technology to improve health outcomes [9].

## E-connecting to Others Who Have the Same Medical Condition

Online support groups exist for almost every disease and condition, and discussion topics within each disease category are limitless. For example, diabetic patients who enjoy scuba diving can learn from fellow diabetic scuba divers how to cope with diabetes 50 feet (15.2 meters) below the water's surface. But just as important as the information exchanged in these e-discussions is the emotional support they provide. For each e-patient seeking a listening "ear," dozens of other patients offer encouragement. In turn, these words of solace are read by hundreds (and sometimes thousands) of other patients who read Internet message boards. This support may be recorded for future reference of patients, clinicians, or health care planners.

## Effect of e-Technology on Health Care Outcomes

Although online intervention may empower patients and may positively affect the patient-physician relationship, a realistic observation is that the Internet will be widely adopted as a part of usual care only if this venue improves patient self-management, betters patient satisfaction, and enhances health outcomes. To determine the success of Internet-based health care, rigorous outcome studies are needed.

A study by McKay et al [10] found that patients who participated in an online diabetes education and support group lowered their blood glucose levels more than controls did. Studies of online support groups for cystic fibrosis patients [11], amyotrophic lateral sclerosis (ALS) patients [12], and single mothers [13] also showed that participants in these online support groups gained satisfaction and confidence in managing their medical condition.

There are limitations to the few studies that have been done. For example, the above study by McKay et al used a small sample, only had short-term follow-up, and there were no solid clinical-outcomes measures. Further research is needed utilizing larger samples over longer periods, controlled and randomized, in tandem with significant outcomes to support policy changes and buy-in efforts for implementation.

## The Achilles' Heel of the Online Revolution

Until recently, the powerful phenomenon of online health care has been largely overlooked by the health care system. Most institutions funding medical research, health policymakers, and health care professionals have ignored both the "e-revolution" and the fact that it is consumer driven. Although the Internet has intuitive potential for improving patient-physician relationships and communication, patient self-management, and health outcomes, outcome research exists for only a few studies and cannot be applied widely because of the studies' limitations.

As a group, physicians themselves have constituted a major source of resistance to online health care. An article, "Why doctors hate the Net" [14], identified 3 specific concerns of physicians:

- E-mail from patients further burdens overflowing physician schedules
- During an already-crowded office visit schedule, e-savvy patients armed with printouts from the Internet waste precious time discussing information from unknown or otherwise-dubious sources
- Much health-related information posted on the Internet is unreliable.

More than a century ago, a similar backlash in health care accompanied introduction of another technology: the telephone. Soon after invention of the telephone by Alexander Graham Bell, much cultural opposition to it was generated by physicians who doubted that the telephone could add value to medical practice. These physicians complained that answering calls would diminish the time available for in-person interaction with patients. Other physicians questioned whether patients would be willing to use the new technology. Some physicians worried that the telephone might destroy the patient-physician relationship [15].

As they did with the telephone, however, physicians are becoming less resistant to using the Internet for delivering patient care. Recent estimates of Internet-equipped physicians vary, but these reports agree that physician adoption of the Internet is increasing noticeably [16], and most agree that physicians (a group sometimes thought technophobic) use the Internet more than do many other sectors of the general adult population [17]. However, physicians have not received information sufficient to convince them that the Internet can help them provide higher-quality care: although 55% of physicians surveyed use e-mail to communicate with professional colleagues, only 13% stated a willingness to send e-mail to patients [18]. In contrast to this finding, 90% of patients surveyed wished to communicate with their physicians by e-mail [19].

In their article "We got mail," Moyer et al [20] highlight issues such as inequity of e-mail access, workload, medical-legal concerns, as well as privacy and confidentiality. Research is necessary to address these issues. Does e-mail from patients really burden a physician's schedule? If so, will triaging by others help? What effect does e-communication have on the patient-physician relationship? Can e-mail reminders from a "virtual case manager" improve health outcomes? Can providers be held liable if an unauthorized third party accesses confidential medical information sent by e-mail? Is e-mail cost-effective?

## Access Gaps

Another obstacle to widely implementing online forms of health intervention is the assumption that lack of necessary technology by many senior, minority, and lower-income patients will exclude them from this intervention. While access to the Internet is less common in these groups, studies show that the "digital divide" is narrowing.

From August 2000 through July 2001, the number of African Americans using the Internet grew nearly 20% [21]. The proportion of wired African Americans (43%), nonetheless, remains low in comparison with the average of online Americans (58%) [21]. Internet access among Hispanics in the United States increased by 25% from March 2000 through February 2001, indicating that more than half of that population is now online [22]. Like African Americans, however, Hispanics have less access to cyberspace than their Caucasian counterparts. In contrast, Asian Americans use the Internet more than other group: more than 75% of that population has access to the Internet [23].

Economics play a part in access. Studies show that lower-income people are less likely to be wired: 37% of those who are not wired have family incomes under $30000, whereas only 18% of those with Internet access have incomes under $30000 [24]. Poor reading skills add even more barriers to those economically disadvantaged for accessing the world of the Web.

The senior population has been slower than other age groups in embracing the Internet but this is changing. A Pew report [25] predicts that with many baby boomers approaching retirement age, seniors' use of the Internet will increase dramatically. The health care industry must be prepared to accommodate this growing segment of the population, many of whom will become homebound but will still need services, training, and reinforcement of medical self-management, as well as continued connection to clinicians and contact with other patients.

While eHealth technologies have the potential to reduce disparities in health care by promoting health and preventing disease, traditionally underserved groups who could benefit the most from eHealth initiatives, are the least likely to have access to such technologies. Although seniors and many minority groups are the fastest-growing segments of new Internet users [22,23,25], we need to better understand access barriers. Furthermore, Eng et al [26] raise important points regarding access issues for those who cannot read at all, those who cannot read English, and those with disabilities.

## Time to " `Byte' the Bullet"

The eHealth care train has not only left the station but is rapidly moving down the track carrying tens of millions of e-patients and many possibilities for transforming patient self-management, improving health outcomes, and enhancing the patient-clinician relationship. Because of substantial opposition to the online revolution, however, the "e-train" has so far evaded the transcontinental health care network.

Fundamental change is needed in our outmoded, Internet-averse system of health care, which still prevails in the United States. The United States health care system must embrace the e-revolution by exploring and taking advantage of the potential benefits of this revolution for improving quality of care. To pursue this goal, rigorous research must explore ways to use e-technology for improving patients' medical self-management, health-related behavior, health outcomes, and relationships with health care practitioners.
